# A BRIEF EXPLORATION OF ARTIFICIAL INTELLIGENCE IN DENTAL HEALTHCARE: A Narrative review

**DOI:** 10.12688/f1000research.140481.1

**Published:** 2024-01-08

**Authors:** Prakrati Kamath, Prathvi Kamath, Sharon J R Saldanha, Thilak B Shetty, Shobha J Rodrigues, Mahesh M, Umesh Y Pai, Puneeth K Hegde, Prashant Bajantri, Sandipan Mukherjee

**Affiliations:** 1 Manipal College Of Dental Sciences, Mangalore, Manipal Academy of Higher Education, Manipal,Karnataka, 576104, India; 2 Department of Prosthodontics, Manipal College Of Dental Sciences, Mangalore, Manipal Academy of Higher Education, Manipal,Karnataka, 576104, India

**Keywords:** Artificial intelligence, Dental Health, Health, Wellness

## Abstract

Artificial intelligence is a computer system which can replicate human behavior and largely supports human actions and interpretation, but not replace human responses. Over the past few decades, the field of artificial intelligence (AI) has experienced phenomenal development and expansion. We are surrounded by several instances of AI. The most typical examples include Chat GPT, Alexa, Google Maps, autocorrect and text editors, e-payments, virtual travel booking agent, social media monitoring, gaming, including chess matches involving computers versus human chess masters, self driving cars, adaptive cruise control, parking assistance, and facial recognition for biometrics such as retinal scans and fingerprint scans. AI has applications in different branches of Dentistry. This review article attempts to highlight these points and lays an emphasis on how AI is driving dentistry in the present and will improve dental care in the future.

A total of 59 papers from an electronic search using Google Scholar and PubMed were used to create this narrative review.

Artificial intelligence can be utilised for diagnosis, decision-making, treatment planning, early detection and prevention of oral disease, and finally result prediction by utilising cutting-edge technology in imaging. It shows how dentists can use it as a useful tool at various phases of clinical cases.

The future of AI in dentistry appears to be outstanding with advancements in full artificial intelligence technology, dental assistance, and dental instructional tools. In order to help dental professionals better grasp AI as a tool to assist their work with enhanced efficiency, investigations need to be done to uncover patterns and foresee future related to oral health concerns.

## Introduction

The field of artificial intelligence (AI) has seen development & growth over past few decades. There are multiple examples of AI, most common would be Chat GPT, Alexa, google maps, autocorrect and text editors, search engine recommendation algorithms, e-payments, virtual travel booking agent, social media monitoring, gaming, self-driving cars, adaptive cruise control, parking assistance, retinal scans and fingerprint scans for biometrics. The purpose of this article is to provide information about the application of artificial intelligence in the field of dentistry that is currently available. ^1^

The phrase “artificial intelligence” was first used by John McCarthy in 1955. ^2^ AI is a branch of applied computer science that endows machines with the ability to mimic intelligent human behaviour. ^3^ An alternate paradigm of limited AI in healthcare, augmented intelligence (AuI), was introduced by the American Medical Association (2018), ^4^ emphasising its support and supplementary function to medical professionals. AuI is increasingly being employed in the dental industry to support clinical and administrative tasks, as a tool to help the clinician complete a task. ^5^

The U.S. Food and Drug Administration (FDA) certified the first robotic surgery system in the country, known as “Yomi,” for dental implant surgery in 2017. Administrative workflows, image analysis, robotic surgery, virtual assistants, and clinical decision support will be the most important AI and AuI applications in the healthcare sector, according to Forbes. ^6^ A 2018 report by Accenture, also included cybersecurity, dose error reduction, and connected machinery. The important domains being connected, according to a 2019 McKinsey report, include cognitive devices, targeted and personalised medicine, robotics-assisted surgery, and electroceuticals The laboratory side of dentistry has been utilising AI/AuI technology for the past 20 years, with the development of chairside scanning along with chairside design and milling of final restorations. ^7^

Physical and virtual AI are both available for the delivery of general healthcare. Physical applications include automatic robotic arms or sophisticated robots. ^8^ Most AI uses are virtual, such software-like algorithms that assist dentists in making clinical decisions. The two main categories of virtual AI approaches are knowledge-based AI and data-driven AI. ^9^

AI is built vertically from self-disclosed concepts used by people to solve problems in an effort to mimic human understanding.

The starting point for machine learning (ML), also known as data-driven AI, is the training of mathematical models utilising data generated by human actions. IBM defines ML as a branch of AI and computer science which focuses on the use of data and algorithms to imitate the way that humans learn, gradually and automatically improving its accuracy. ^10^

There are three types of data-driven learning: supervised, unsupervised, and semisupervised. On the supervised platform, algorithms learn the correlations between data instances and labels using manually labelled training data sets, producing the desired and known results ^.^ In Supervised learning, ML algorithms used are Support vector machines (SVMs), decision tree (DT), random forest (RF), and artificial neural networks (ANNs). ^11^

Algorithms are given unlabeled data in unsupervised learning, where they must recognise hidden data patterns that researchers may not have thought of as yet, producing unknown results. Principal component analysis and k-means clustering are common methods used in unsupervised learning. ^12^

Semisupervised learning creates the predictive model by learning from a sizable set of unlabeled training examples and a constrained set of labelled training examples. ^13^

Deep learning (DL), also known as Deep Neural Networks, is a subset of Machine Learning. Image classification, detection, and segmentation have all been accomplished using deep learning. In a medical setting, function of DL might take a variety of clinical data as input and return proof for a certain medical condition. ^11^

So far the most popular and successful DL architecture in dentistry is convolutional neural networks (CNN) for image analysis. They combine spatial data and picture configuration from 2D and 3D photos. CNNs are frequently employed in medical applications, such as drug research, diagnosis, treatment, and related procedures. ^14^

Intelligent document processing (IDP) and natural language processing (NLP) use AI/AuI to recognise concepts in written and spoken language, record these concepts as digital data elements, and interact with system users in natural language. The term “cognitive computing” describes the practise of simulating human thought processes using computer technology. Deep learning is used by computer vision to identify patterns in images and videos.

Clinical decision support (CDS) tools and technologies assist clinical teams by taking over some tedious activities, alerting them to potential issues, or making recommendations that the clinical team and patient should take into account. Typically, a training dataset consists of a number of dental pictures, such as intraoral radiographs. ^15^

Validation datasets are obtained by testing against a dataset of test cases, that serve as the industry's gold standard. After an algorithm is created using the training dataset and validation dataset, the testing dataset may be used to verify the algorithm’s ability to perform on new data. For supervised learning approaches, the accuracy of the training set's classification is referred to as ground truth (also known as gold standard classification). This is used to support or refute research hypotheses in statistical models. Accuracy of measurement and comprehensiveness are dependent on the expertise, instruments, and timing of the clinician.

## Methods

A total of 59 papers from an electronic search using Google Scholar and PubMed were used to create this narrative review.

## Results

### Applications In Medical-Aided Diagnosis Dental Radiology

A clinician's evaluation of a patient's symptoms, results of diagnostic tests, and other data are used to make a diagnosis for a particular ailment. The clinical outcomes will be more precise and effective with the integration of AI into the current dentistry clinical workflow. ^16^ Computerised interpretations may be based only on radiologic image analysis or may integrate data from past, present, and future laboratory and clinical findings.

The type I data, such as radiographic results or cytopathologic images, is the focus of the prospective clinical use of AI/AuI in dentomaxillofacial radiologic imaging.

AI's clinical promise depends on its capacity to identify important anatomical structures by labelling and/or segmenting them. The parotid gland, mandibular canal, interdigitated tongue muscles, and nerves are a few examples. While the discrepancy between expert measurement of anatomic location and AI-based segmentation is only in the millimetre range, ^17^ it is nonetheless significant in case of structures like neurovascular canals,if missed could result in serious surgical difficulties.

According to Yilmaz *et al* ^18^ SVM in cone beam computed tomography (CBCT) has demonstrated accuracy in separating periapical cysts from keratocystic odontogenic tumours. Interpreting radiologic disease signs, such as calculus identification, marginal discrepancies, apical periodontal inflammation, detection of odontogenic cysts, tumours, as well as screening for possible diseases including osteoporosis.

It has also been demonstrated that AI/AuI techniques can help with early oral cancer identification. ^19^ The benefit is that it may contribute to lowering the mortality rate linked to delayed or missed diagnosis. ML-based predictive models including all patient performance variables,for oral cancer prognosis have been developed, A brush biopsy and cytology-based risk classification model created using ML techniques like SVM and RF. In order to predict occult nodal metastases, decision forest, SVM, and gradient boosting algorithms were used. ^20^ Of the algorithms DT, SVM, decision forest, and naive Bayes in predicting loco regional recurrence of squamous cell carcinoma; these algorithms beat a current clinical model that solely relied on tumour invasion depth. ^21^ Moreoral robotic surgery has gathered a lot of attention for oropharyngeal cancer surgery. ^22^

Clinical data-driven decision trees assist in deciding which imaging test is best to perform in order to determine whether surgery is necessary, the type of surgery that should be performed, and the results of the operation, for example in orthognathic surgery situations. After the removal of impacted mandibular third teeth, the ANN was successful in predicting facial edema. (Zhang *et al*. 2018). ^23^

In order to improve the speech comprehension of oral surgery patients during postoperative rehabilitation, an ML-based voice conversion technique has been used. ^24^ In order to enhance results following a cleft lip operation, Microsoft and Operation Smile are developing a tool that leverages AI to standardise patient before-and-after pictures.

Although there are currently just a few uses of AI/AuI in oral and maxillofacial surgery, this may change in the future.

### Temporomandibular Joint Disorder

By including patient complaints, clinical and biochemical markers, and objective radiomic properties in training data sets and accumulating larger samples of the aforementioned data sets, a computer-assisted diagnosis technique is necessary to improve diagnostic accuracy of temporomandibular joint disorders (TMD). It is possible to identify TMD’s based on a patient's complaint and medical background. A model based on natural language processing was successful in distinguishing between diseases that mimic TMDs and actual TMDs based on the amount of words used in the patient's primary complaint and the size of the mouth opening. ^25^ According to Shukri *et al.* (2019), classification of condylar morphology showed conformity using ANNs based on CBCT. Internal derangements can be correctly predicted using AI/AuI-assisted imaging. The design of nightguards employing AI/AuI-based technology in conjunction with intraoral scanning ensures that the patient bites properly.

### Dental Implant and Prosthetic Use

Since 1980, dental implants have been widely utilised to restore lost teeth. In order to identify the implant type in periapical and panoramic radiographs, numerous AI/augmented intelligence models have been constructed. Three AI models using panoramic and radiographic images have been proposed as trustworthy methods for assessing the success of osteointegration, as well as for optimising dental implant design and for spotting fractured dental implants. ^26^ On most dental design software, a digital AI-based wax-up can be finished in a matter of seconds. It can help with the creation of occlusal guards and surgical guides. The chairside use of CAD/CAM is enhanced by AI integration. ^27^ The most recent AI/AuI-based laboratory technology includes automated margin marking, case routing, and scoring.

An ablation system integrating robotics and a picosecond laser has been developed for abutment tooth preparation. The average linear ablation errors of this technology, according to in vitro studies, were just 0.06 mm for wax resin and 0.05 mm for dentin. ^28^ However, the 3.5-hour dentin ablation time prevented its use in clinical settings. In the field of fixed prosthodontics, research on an autonomous robotic system is still in its infancy. A clinical decision support model for removable prosthodontics that uses ontology and case-based reasoning was able to suggest a design for customised removable partial dentures. ^29^

Imaging combined with several AI/AuI technologies is already assisting with morphological studies, OSA classification, screening, and automated landmark documentation. ^6^ Future applications could involve better risk detection, remote monitoring, and choosing the patient's best course of treatment.

### Periodontic Use

Periodontitis varies in its distribution and severity. A periodontal probe and radiographic images are frequently used to collect information in order to establish the diagnosis, prognosis as well as the available treatment options. Radiographic bone level and clinical attachment levels (CAL) have been calculated using AI/AuI evolutions. ^30^ The alveolar bone level on panoramic or intraoral radiographs has been measured in research using deep learning techniques. Current radiography pictures might not be sensitive enough to detect the earliest stage of bone loss, even if they are of high quality.

Although the relationship between immunologic responses, microbial composition and the aetiology of periodontitis has not yet been fully clarified, artificial intelligence-based classifiers can distinguish between aggressive and chronic periodontitis. An SVM that focuses on the relative bacterial load is one example, as is a multilayer perceptron neural network that concentrates on leukocytes, interleukins, and IgG antibody titers. ^31^ A validated periodontal diagnosis is aided by artificial intelligence by including conventional symptoms, immunologic, and microbiological components.

### Orthodontics

A great amount of research has been done in the field of orthodontics on growth evaluation, a crucial component in choosing the best course of treatment. The use of hand-wrist radiographs and the cervical vertebrae maturation (CVM) detected on the lateral cephalogram are currently two of the most popular techniques for measuring growth. ^32^ The classification of cervical vertebral maturation stage and vertebral body shape by ANN and DT, respectively, produced the best results. ^33^ Using ML techniques such ANN, SVM, RF, and DT, a diagnostic model based on lateral cephalometric radiographs was developed to measure cervical vertebral maturation. ^34^

Convoluted Neural Network powered AI/AuI system analysis showed a tracing accuracy of over 90% and came to the conclusion that automated identification was more trustworthy than manual identification. ^35^ In the future, more precise and accurate automated cephalometric analysis should be possible thanks to hybrid methodologies that combine ML and knowledge-based algorithms.

AI/AuI technologies for facial analysis increase the precision of diagnosis, case formulation, and treatment planning. By identifying certain face characteristics, CNNs were able to estimate apparent age and score attractiveness. ^36^ Scans, model design, and retainer manufacture are three areas where AI/AuI assisted technologies are revolutionising orthodontic office therapy and care practises.

In the diagnosis of orthodontic treatment needs, the Bayesian network had excellent agreement with orthodontists. ^37^ The two potential uses of AI in orthodontics are result grading and treatment need assessment. ^38^

### Caries Detection Use

The detection and diagnosis of signs including caries are frequent in dentistry. Early enamel caries detection may improve minimally invasive methods for treating early caries. From a clinical perspective, Imaging techniques such radiograph, optical coherence tomography, quantitative light-induced fluorescence, intraoral scanning, or near-infrared light transillumination are used in addition to visual-tactile detection. Dental X-rays or images from near-infrared light transillumination can be utilised to diagnose caries using deep learning and convolutional neural networks (CNNs). From near-infrared transillumination images, DL found proximal carious lesions. ^39^ When applied to assess patient demographic, nutritional, lifestyle, and clinical data, SVM performed at its peak level. ^40^ Predictive models based on ML methods like SVM, RF, and k-nearest neighbours were used to pinpoint those who are more prone to develop dental surface loss and root cavities.

According to a study from 2021, ^41^ AI-enabled sensitivity was shown to be present in enamel caries, but not in early or advanced dentin lesions. None of the dental imaging methods that are now available can tell the difference between active and halted caries. Based on an encoder-decoder architecture (U-Net), DL divided CBCT voxels into categories such as “lesion,” “tooth structure,” “bone,” “restorative materials,” and “background,” producing results that were comparable to those of medical professionals in the diagnosis of periapical lesions. ^42^ DL with CNN has supplanted other AI components in cariology and endodontic diagnostics due to its capacity for automated lesion segmentation. ^43^

The ability to offer unbiased baseline and follow-up measurements for remineralization is an attractive prospect for AI/AuI. AI/AuI's capability to calculate the percentage of demineralization and look at patterns may help demonstrate the validity of longitudinal data assessment over time as opposed to a one-time, cross-sectional examination. Additionally, when employing clinical pictures as a potential machine-readable source of information for diagnostic purposes, additional pathological features, such as developmental flaws or dental restorations, must also be taken into account.

### Endodontic Use

For diagnosis of radiolucent jaw lesions like apical periodontitis, which affects around 75% of endodontic patients panoramic and intraoral periapical radiographs are employed. ^44^ Dental history, clinical data, and sociodemographic information, for example, can all be connected using AI. Digital radiography, cone beam computed tomography, computer-aided diagnostics (CAD), 3D printing, and guided endodontics are the various diagnostic methods utilised in endodontics.

Using 83 features from the “Endodontic Case Difficulty Assessment Form” of the American Association of Endodontists as input, SVM and ANN were able to predict the degree of difficulty of cases requiring root canal therapy with >90% accuracy. ^4^ This demonstrated how ML might potentially help general dentists make better decisions when referring patients to endodontists. ^45^

Probabilistic neural networks (PNN), Convoluted Neural Network and Machine Learning systems appear to be the focus of recent breakthroughs in the use of AI/AuI systems in endodontics. ^46^ Identification - A diagnostic aid that aids in the identification of periapical lesions, crown and root fractures, the apical foramen, and the efficacy of an existing root canal filling. It is utilised to provide information on the morphology of the root and root canal systems as well as the degree of canal curvature.

Three commonly used methods for evaluating root canal length are hand sensation, radiological determination, and use of an electronic apex locator. Modern technologies for locating the apical foramen include CBCT and computerised apex locators. Due to its capability to do automatic lesion segmentation, the DL with CNN has grown to be the most popular AI component utilised in endodontic diagnostics. The DL system of AI is also used to assess the root canal morphology. The ANN diagnosis method results in a better radiographic estimation of working length and helps to enhance diagnosis. Both intact and non-intact teeth, that have undergone endodontic treatment can benefit from the use of CBCT in the diagnosis of vertical root fractures (VRF). Campo *et al* ^47^ described the case-based reasoning (CBR) paradigm to reasonably estimate nonsurgical endodontic retreatment outcomes and the advantages and dangers. AI is being utilised to forecast stem cell viability and the results of endodontic retreatment.

### Mucosal Lesions

In order to distinguish oral lichen planus from other white lesions of the oral mucosa, ^48^ found that the expression of the inflammatory cytokine genes ANN, SVM, and RF can be used. By spotting steatosis (i.e., abnormal retention of lipids) of the salivary gland parenchyma in ultrasound pictures, ANN was found to be more effective at differentiating between people with xerostomia and those with true Sjögren's syndrome. ^49^

### Preventative and Maintenance Use

Clinical assessments, patient dental histories, and interpretation of diagnostic radiographs are used for preventive restoration treatment. The intention is to discover and measure the radiographic extent of caries in order to possibly establish the least invasive and most efficient course of treatment. It can also help in the early discovery of issues such as dental impaction, congenitally missing teeth, ectopic eruption, ankylosis of a primary tooth, loss of space inside the dental arch, and other atypical dental morphology.

### Disease Prediction and Prognosis

Classifier and predictive AI models, which explore connections between clinical symptom-related features and patient data, assist in identifying risk factors and forecasting long-term consequences of dental illnesse. A patient list that comprises the patient's ongoing needs and health details can be scheduled by AI. If patient records are made public, it could be able to anticipate patient-specific drug problems. AI could aid in diagnosis, staging, and outcome prediction. ^6^

A multilayer perceptron neural network successfully predicted tooth mobility and the durability of resin composite restorations based on the characteristics of the patients. According to Yamaguchi *et al* ^50^ CNNs were successful in accurately predicting the likelihood of de-bonding of CAD/CAM resin composite crowns. A prediction model combining ANN could be used in the future to assess regenerative dentistry methods in a simulated inflammatory microenvironment. Great sensitivity in detecting oral malignant (93%) and high-grade prospective malignant (73%) lesions by using CNN to score the malignancy of cytology pictures acquired from a telemedicine platform. ^9^

Applications of virtual AI hold enormous promise for streamlining clinical procedures. They have been instrumental in the growth of precision dentistry.

## Limitations of Artificial Intelligence in Dentistry

The inability to represent internal decision-making processes, the limitations of classical computing, the disregard for moral principles in the design of AI frameworks, the lack of data curation, sharing, and readability are all potential obstacles that could prevent the routine implementation of AI.

### Data Acquisition

Limitations such inadequate data sharing and curation, ^51^ a lack of knowledge about data processing, measurement, and validation. ^52^

### Interpretability

Interpretability is significant for two reasons. First, check to see if the algorithm makes sense in terms of how humans and technology interact. Second, because there is a lack of transparency and interpretability, it is difficult to predict failures and generalise specific techniques for situations that are comparable. ^53^

### Computing Power

Processing power must always be upgraded for AI applications. In comparison to conventional methods, quantum computing processes are tenfold faster. ^54^ Quantum computing is a great platform for accelerating ML, ^55^ from algorithms to data collecting and modelling due to its capacity to process data concurrently from widely diverse data sets.

### Ethical Considerations

According to Currie G, ^56^ the doctor is responsible for each patient and how the data is used. AI is not accountable whether it is deployed in a supervised or unsupervised environment. Determining legal obligation raises an ethical dilemma. When the distinction between human responsibility and AI-based diagnosis based on chatbots is becoming increasingly blurry, it is irrational to impose the same social and ethical criteria that apply to people. ^57^ Ethical contradictions show the need for clear guidelines on how AI should be applied in healthcare settings.

When AI/AuI technologies are integrated into EDR, other criteria, including security, privacy, trust, quality, safety, and data standardisation, will ultimately determine the validity and utility of these dental technologies. Additionally, consumers should anticipate having access to their EDR digital data, including images, in the near future as required by the federal laws regarding information blocking included in the 21st Century Cures Act.

### Impact of AI on Dentists

There are questions over whether AI will ever replace dentists.

The knowledge of dentists is required for higher-level comprehension since machines cannot provide clinical intuition, intangible perception, or empathy, which are necessary for providing customised healthcare and professionalism. Modern AI excels in two areas: the utilisation of codified knowledge and the extraction of understanding from vast amounts of data. ^6^ It cannot build associations, though, and in a therapeutic situation, it is only partially capable of making complex decisions. It is impossible to directly transform the most exciting part of human-to-human conversation into computer language.

The development of AI and dental education should go hand in hand. When patients cannot access quick dental care, teledentistry offers a more modern technique that could help them manage oral problems. ^58^ Images taken on cellphones are helpful tools when information are gathered through distant dialogues or internet platforms to assess the clinical conditions. Furthermore, a lot of dental clinics have digital records of their patients, and these already-existing photos might help the physician with diagnosis and treatment planning. It may also be used to keep track of treatment compliance and development.

Image quality and quality analysis are predicted to significantly increase as a result of artefact reduction, low-dose imaging, and automated analysis of 2D and 3D image datasets to identify artefacts and technical errors and less radiation exposure. Over time, the suppliers' selections of visualisations, designs, or essential tools will evolve. It is advantageous for the laboratory industry to integrate AI/AUI based technology into their ecosystem because of the benefits of consistency, repeatability, cost savings, time, and enhanced intelligence.

By developing a comprehensive open-access standard data set that contains information on demographics, clinical trials, and treatments, thereby increasing the amount, quality, and readability of data by standardising data curation and reporting technique, Dental professionals will have access to more organised documentation alternatives and evidence-based guidelines.

## Conclusions

AI is quickly evolving and has applications in diagnosis, therapy, and prognosis. AI could be used as an augmentation tool by dental practitioners to assist them in performing more valuable tasks, such as integrating patient data and improving professional interactions. ^59^ AI is recognised as a useful tool for dentists, despite the difficulties that must be overcome in terms of data collecting, interpretation, computing capacity, and ethical issues.

With careful design and extensive clinical validation, AI may be user-friendly, transparent, repeatable, and impartial. Future AI development should continue to put the needs of people first while enhancing its capacity to handle massive amounts of data.

In order to support human-technology rapport and ensure AI'S affirmative development, which will revolutionise dentistry practise, it is imperative to have a proactive attitude towards AI.

## Data Availability

Reporting guidelines Figshare. A BRIEF EXPLORATION OF ARTIFICIAL INTELLIGENCE IN DENTAL HEALTHCARE.xlsx DOI: https://doi.org/10.6084/m9.figshare.23803026 This project contains the following underlying data
• A BRIEF EXPLORATION OF ARTIFICIAL INTELLIGENCE IN DENTAL HEALTHCARE.xlsx (Details of included studies in the present narrative review) Data are available under the terms of the Creative Commons Zero “No rights reserved” data waiver (CC0 Public domain dedication).
