# Supplementary material for: A brief exploration of artificial intelligence in dental healthcare: a narrative review
Source: F1000Res. 2024 Apr 8;13:37. Originally published 2024 Jan 8. [Version 2] doi: 10.12688/f1000research.140481.2 (PMC12361878; doi:10.12688/f1000research.140481.2)
Supplement: Peer review history

# Reviewer report: Reviewer response for version 2

**DOI:** 10.5256/f1000research.164552.r379075

**Published:** 2026-08-14

**Authors:** Omid Panahi

**Affiliations:** 1 Faculty of Dentistry, Centro Escolar University, Manila, Philippines

This review addresses a highly relevant and rapidly evolving topic at the intersection of artificial intelligence and implementation science. The article provides a broad overview of current developments, opportunities, and challenges associated with the use of AI to support the implementation of evidence-based interventions. The topic is discussed in a generally comprehensive manner, and the manuscript successfully highlights key concepts, methodological considerations, and future directions for the field.

 The review is well organized and written in clear, accessible language, making it suitable for a multidisciplinary audience that includes researchers, clinicians, implementation scientists, and policymakers. The conclusions are balanced and appropriately reflect the current state of the literature.

 Most factual statements are adequately supported by references. However, given the rapid pace of development in AI, the authors may wish to strengthen several sections by incorporating additional recent literature, particularly regarding large language models, generative AI applications, responsible AI frameworks, and emerging implementation strategies. Expanding the discussion of recent evidence would further enhance the currency and completeness of the review.

 Minor revisions that could improve the manuscript include:

 (1) updating selected references with the most recent studies,

 (2) providing a more explicit discussion of ethical, regulatory, and equity considerations related to AI implementation, and

 (3) highlighting areas where evidence remains limited and future research is needed.

 Overall, this is a valuable and informative review that makes a useful contribution to the literature. The manuscript is scientifically sound, clearly written, and suitable for indexing after minor revisions.

Is the review written in accessible language?

Yes

Are all factual statements correct and adequately supported by citations?

Partly

Are the conclusions drawn appropriate in the context of the current research literature?

Yes

Is the topic of the review discussed comprehensively in the context of the current literature?

Yes

Reviewer Expertise:

Ai in Dentistry, Digital Dentistry. Oral implantology

I confirm that I have read this submission and believe that I have an appropriate level of expertise to confirm that it is of an acceptable scientific standard, however I have significant reservations, as outlined above.

# Reviewer report: Reviewer response for version 2

**DOI:** 10.5256/f1000research.164552.r401441

**Published:** 2025-08-18

**Authors:** Sreelatha S V, Vimal Kumar Karnaker

**Affiliations:** 1 AB Shetty Memorial Institute of Dental Sciences, NITTE (Deemed to be University), Mangalore, India; 2 Microbiology, NITTE KS Hegde Medical Academy (Ringgold ID: 29220), Mangalore, Karnataka, India

This manuscript offers a timely and pertinent overview of the applications of artificial intelligence (AI) in dental healthcare. It is well-referenced, written in accessible language, and reflects commendable effort in synthesizing current literature.

 The manuscript provides a well-structured summary of existing literature, effectively addressing the benefits, limitations, and real-world applicability of AI tools in dentistry. It thoughtfully discusses key challenges such as data standardization, algorithmic bias, and practitioner acceptance. The use of thematic subheadings aids in mapping AI’s role across various dental specialties.

 The conclusion has been appropriately extended to incorporate a future-oriented outlook, addressing implementation barriers, clinical recommendations, and potential directions for further research.

 The manuscript maintains an appropriate academic tone and is generally concise.

 The references appear consistent with the journal’s prescribed citation style. I am satisfied with the content and presentation of the manuscript and approve its indexing.

Is the review written in accessible language?

Yes

Are all factual statements correct and adequately supported by citations?

Yes

Are the conclusions drawn appropriate in the context of the current research literature?

Yes

Is the topic of the review discussed comprehensively in the context of the current literature?

Yes

Reviewer Expertise:

Oral Oncology, Oral infections

We confirm that we have read this submission and believe that we have an appropriate level of expertise to confirm that it is of an acceptable scientific standard.

# Response

**Published:** 2025-08-18

**Authors:** Sharon Saldanha

**Affiliations:** PROSTHODONTICS, Manipal Academy of Higher Education, Manipal, Karnataka, India

Respected Reviewer,

 We sincerely thank you for your thoughtful and encouraging feedback on our manuscript. We are grateful for your positive appraisal of the structure, language, referencing, and the overall synthesis of the current literature on the applications of artificial intelligence in dental healthcare.

 Your acknowledgement of our efforts to highlight the benefits, limitations, and applications  of AI tools, as well as  critical challenges , is truly motivating. We are pleased that the use of thematic subheadings and the extended conclusion, addressing clinical recommendations and future directions, were found to be effective. We are particularly encouraged by your note on the appropriateness of the academic tone, conciseness, and consistency of references. 

 We thank you once again for your valuable time and supportive remarks, which will inspire us to continue contributing quality research in this evolving field.

 With sincere regards,

 Sharon Saldanha

 On behalf of all co-authors

# Reviewer report: Reviewer response for version 2

**DOI:** 10.5256/f1000research.164552.r304284

**Published:** 2024-09-26

**Authors:** Snigdha Pattanaik

**Affiliations:** 1 University of Sharjah, Sharjah, Sharjah, United Arab Emirates

The manuscript is very well researched & adequately referenced. It accurately depicts the findings of 59 papers that have been written on Artificial Intelligence (AI) and draws inference from the same.  It underlines the use of AI for dental professionals to better facilitate their grasp on the same as applicable to the field of dentistry for present concerns & improvements in the future.

Is the review written in accessible language?

Yes

Are all factual statements correct and adequately supported by citations?

Yes

Are the conclusions drawn appropriate in the context of the current research literature?

Yes

Is the topic of the review discussed comprehensively in the context of the current literature?

Yes

Reviewer Expertise:

orthodontics

I confirm that I have read this submission and believe that I have an appropriate level of expertise to confirm that it is of an acceptable scientific standard.

# Reviewer report: Reviewer response for version 1

**DOI:** 10.5256/f1000research.153839.r253468

**Published:** 2024-03-27

**Authors:** Hao Ding

**Affiliations:** 1 The University of Hong Kong, Hong Kong, Hong Kong

Dear Authors,

 The aim of this study is to provide readers with a general overview of the current state of artificial intelligence (AI) in dentistry. As AI is still rapidly evolving, an updated review on the subject matter is welcomed. The topic is interesting and will be useful for dental clinicians, computer engineers, and other interested parties. While the topic fits within the scope of the journal, there are some concerns that need to be addressed. Please see my comments below:

 General Comments:

 1. Given that there have been many review articles published in this field, I would suggest acknowledging existing review articles in the field and discussing the emphasis of the current review.

 2. Consider combining some of the short paragraphs to improve the flow of the manuscript.

 Abstract:

 1. Consider revising the Abstract. The first paragraph contains too much information that is not related to dental AI, while the third and fourth paragraphs about dental AI are too brief.

 2. Move the sentence "This review article attempts to highlight these points and lays emphasis on how AI is driving dentistry in the present and will improve dental care in the future" to the end of the abstract.

 Introduction:

 1. In the third paragraph of the Introduction, it would be clearer if the authors can clarify that robotic surgery is not considered as AI, as they are two different concepts.

 Methods:

 1. The Methods section appears to be too brief. I recommend adding information such as the keywords used, selection criteria employed, publication year range, and other relevant details when searching for reference articles.

 Results:

 1. I personally think it would be more appropriate to change the subtitle from "Results" to "Discussion". As for a review paper, a discussion section is needed to synthesize all the information retrieved from the literature search into comprehensive paragraphs. This is where readers should find the information they are looking for in one location.

 2. I suggest merging the "Limitations of Artificial Intelligence in Dentistry" section into the discussion part to simplify the structure of the manuscript.

 3. I recommend including tables of information to make it easier for readers to access and understand the review. The tables can include specific AI tasks, input data types and dataset sizes, types of algorithms used, and results of the studies.

 4. It would be beneficial to include studies on Generative AI in dentistry.

 Thank you for considering these comments. I believe that addressing these concerns will strengthen the scientific quality of your manuscript.

 Best regards,

 Ding Hao

Is the review written in accessible language?

Yes

Are all factual statements correct and adequately supported by citations?

Yes

Are the conclusions drawn appropriate in the context of the current research literature?

Yes

Is the topic of the review discussed comprehensively in the context of the current literature?

Partly

Reviewer Expertise:

Digital Dentistry, Dental Materials

I confirm that I have read this submission and believe that I have an appropriate level of expertise to confirm that it is of an acceptable scientific standard, however I have significant reservations, as outlined above.
